# A single-center, phase 1/2a trial of hESC-derived mesenchymal stem cells (MR-MC-01) for safety and efficacy in interstitial cystitis patients

**DOI:** 10.1093/stcltm/szaf018

**Published:** 2025-05-19

**Authors:** Yoon Soo Kyung, Ki-Sung Hong, Hyung-Min Chung, Jung Hyun Shin, Myung-Soo Choo, Eun-Young Kim, Jeong Min Shin, Ah Reum Kang, Jin Won Seo, Juhyun Park, Se-Pill Park

**Affiliations:** Department of Urology, Asan Medical Center, University of Ulsan College of Medicine, Seoul 05505, Korea; Health Screening and Promotion Center, Asan Medical Center, University of Ulsan College of Medicine, Seoul 05505, Korea; Mirae Cell Bio Co., Ltd., Seoul 04795, Korea; Mirae Cell Bio Co., Ltd., Seoul 04795, Korea; Department of Stem Cell Biology, School of Medicine, Konkuk University, Seoul 05029, Korea; Department of Urology, Ewha Womans University, Mokdong Hospital, Seoul 07985, Korea; Department of Urology, Asan Medical Center, University of Ulsan College of Medicine, Seoul 05505, Korea; Doctor Joo Urology Clinic, Seoul 06060, Korea; Mirae Cell Bio Co., Ltd., Seoul 04795, Korea; Mirae Cell Bio Co., Ltd., Seoul 04795, Korea; Mirae Cell Bio Co., Ltd., Seoul 04795, Korea; Mirae Cell Bio Co., Ltd., Seoul 04795, Korea; Department of Urology, Asan Medical Center, University of Ulsan College of Medicine, Seoul 05505, Korea; Mirae Cell Bio Co., Ltd., Seoul 04795, Korea; Department of Bio Medical Informatics, College of Applied Life Sciences, Jeju National University, Jeju 63243, Korea

**Keywords:** clinical trial, embryonic stem cell, Hunner lesion, interstitial cystitis, mesenchymal stem cell

## Abstract

This study investigated the safety and efficacy of MR-MC-01, a mesenchymal stem cell therapy derived from human embryonic stem cells, in patients with interstitial cystitis (IC), particularly those with Hunner lesions unresponsive to pentosan polysulfate sodium (PPS). Conducted as a prospective, randomized, double-blind, placebo-controlled phase I/IIa clinical trial, it enrolled 22 patients, with six completing phase I and 16 participating in phase IIa. Phase I tested 2 doses (2.0 × 10^7^ and 5.0 × 10^7^ cells) to determine the maximum tolerated dose (MTD), revealing no dose-limiting toxicities and only mild adverse events such as transient hemorrhage and bladder pain.

In phase IIa, 12 participants received the MTD of 5.0 × 10^7^ cells, and 4 received placebo. Significant reductions in interstitial cystitis questionnaire (ICQ) and pain urgency frequency (PUF) scores were observed in the treatment group. Improvements were noted in nocturnal voiding frequency and Hunner lesion size, with 8 patients showing either a reduction or complete resolution of lesions after 6 months. The global response assessment (GRA) reported moderate to marked improvement in 41.67% of treated patients versus 25% in the placebo group.

MR-MC-01 demonstrated no serious drug-related adverse events, highlighting its favorable safety profile. These findings suggest that MR-MC-01 not only alleviates symptoms but also promotes structural recovery in IC, making it a promising treatment option. Further large-scale, long-term studies are warranted to confirm these results and optimize therapeutic protocols. (Identifier: NCT04610359)

## Graphical Abstract

**Graphical Abstract:**
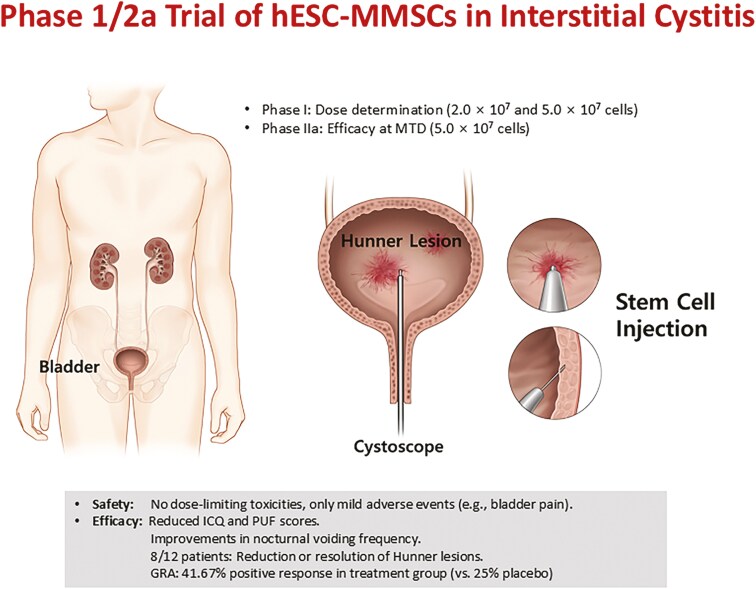

**Significance statement**

Interstitial cystitis (IC), particularly the Hunner-type, is a chronic, debilitating condition with limited effective treatments. This first-in-human, double-blind, placebo-controlled clinical trial demonstrated that MR-MC-01, a human embryonic stem cell-derived mesenchymal stem cell therapy, is safe and shows promising efficacy in patients with refractory IC. MR-MC-01 not only alleviated clinical symptoms but also induced structural improvements in Hunner lesions. These findings highlight its potential as a regenerative treatment option that goes beyond symptom control, offering a novel approach to modifying disease progression in IC.

## Introduction

Interstitial cystitis (IC) is a chronic urological condition marked by pelvic pain, urinary urgency, frequency, and nocturia, which significantly affects patients’ quality of life.^1^ The etiology and pathophysiology of IC remain uncertain, which makes the condition particularly challenging to treat effectively.^2,3^ Current therapeutic options, such as oral medications,^4,5^ bladder instillations,^6^ nerve stimulation,^7^ and transurethral fulguration,^8,9^ primarily focus on symptom management. However, these treatments often provide only temporary relief,^10-12^ leading to high recurrence rates and leaving many patients with ongoing pain and discomfort.^13^ This underscores the urgent need for treatments that not only relieve symptoms but also target the underlying pathophysiological mechanisms of IC.

Stem cell therapy represents a promising new avenue in regenerative medicine. Mesenchymal stem cells (MSCs), in particular, have shown significant immunomodulatory and regenerative potential.^14,15^ MR-MC-01, derived from human embryonic stem cells (hESCs), stands out due to its superior ability to repair damaged tissue and modulate inflammation more effectively than adult-derived MSCs. This makes MR-MC-01 a strong candidate for treating conditions like IC, which involve chronic tissue injury and immune dysregulation.^16-20^

Preclinical studies have demonstrated that MR-MC-01 reduces inflammation, promotes angiogenesis, and supports tissue repair in damaged bladder tissue. Initial safety data from phase I trials revealed no significant adverse events, with promising improvements in bladder function and pain relief.^19^ These results suggest that MR-MC-01 could address not only IC symptoms but also the underlying inflammatory and tissue damage processes, positioning it as a potential comprehensive treatment option.

Despite these promising results, there is limited clinical evidence supporting the effectiveness of stem cell therapies in IC, particularly for those with Hunner lesions. Previous studies have explored the use of stem cells for inflammatory and autoimmune diseases, but MSCs for IC remain under-researched.^21,22^ This gap emphasizes the need for clinical trials to evaluate the safety and efficacy of stem cell-based therapies like MR-MC-01 in treating IC, particularly in those with Hunner lesions who have not responded to conventional treatments.

## Material and methods

### Patient selection

This randomized, double-blind, placebo-controlled phase I/IIa study was conducted at a single center from 2022 to 2023. Participants had interstitial cystitis with Hunner lesions and persistent symptoms for over 6 weeks, refractory to treatment with pentosan polysulfate sodium (PPS). Inclusion criteria required a minimum score of 4 on the Visual Analogue Scale (VAS), 12 on the Interstitial Cystitis Questionnaire (ICQ), and 13 on the Pain, Urgency, Frequency (PUF) scale.

The study was conducted in accordance with the Declaration of Helsinki and Good Clinical Practice (GCP) guidelines. The clinical protocol was approved by the Institutional Review Board of Asan Medical Center (IRB No. 2022-0955) and registered at ClinicalTrials.gov (Identifier: NCT04610359). Written informed consent was obtained from all participants prior to enrollment.

### Study design

The study aimed to evaluate the safety and efficacy of MR-MC-01 administered via submucosal injection into the bladder. It was divided into 2 phases: Phase I to determine dose-limiting toxicities (DLT) and the maximum tolerated dose (MTD), and phase IIa to explore safety and preliminary efficacy. This study was a prospective, randomized, double-blind, placebo-controlled phase I/IIa trial registered at ClinicalTrials.gov (Identifier: NCT04610359).

**Figure 1.**
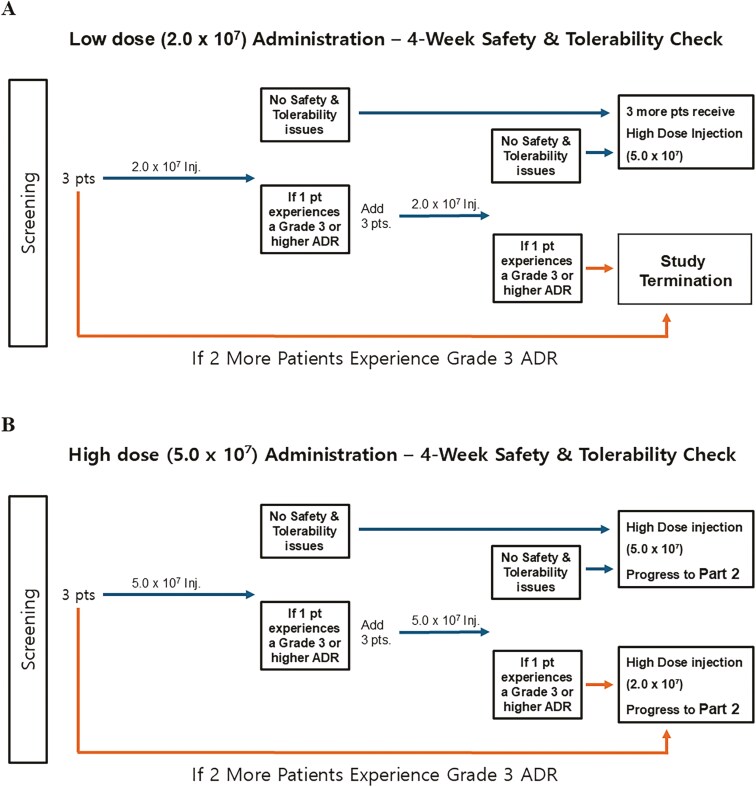
Phase I: dose-limiting toxicities and maximum tolerated dose. (A) Low dose administration: Three participants received a low dose of MR-MC-01 at 2.0 × 10^7^ cells. Dose-limiting toxicities (DLTs) were defined as grade 3 or higher adverse events. If no DLTs occurred during a 28-day follow-up, the trial could proceed to the next stage. (B) Following confirmation of safety, the trial advanced to a high dose of MR-MC-01 at 5.0 × 10^7^ cells. This high dose was established as the maximum tolerated dose (MTD) for the Phase IIa trial

In phase I, 3 participants received a low dose of 2.0 × 10^7^ cells. If no grade 3 or higher adverse events were observed during a 28-day follow-up, the next cohort received a higher dose of 5.0 × 10^7^ cells. The high dose was determined to be the MTD and used in phase IIa (Figure 1). In this phase, 12 participants received M-MC-01 and 4 received placebo, following a 3:1 randomization ratio. Follow-up visits were scheduled at 1, 3, and 6 months post-administration.

### Randomization and blinding

In phase IIa, both MR-MC-01 and placebo were used in a double-blind manner. Randomization was carried out using a computer-generated table to assign participants to treatment or control groups. The 3:1 treatment-to-placebo ratio maximized data collection while maintaining statistical rigor. Confidentiality of treatment assignments was ensured throughout the study.

### Intervention

MR-MC-01 was prepared and cryopreserved at a certified facility, thawed, and administered via submucosal injection into 20 sites in the bladder wall around Hunner lesions using a cystoscope. The placebo group received an equal volume of saline in the same manner to ensure blinding. A total of 10 cc was injected per participant. The use of placebo was essential to control for potential psychological or physiological responses.

### Safety assessment

Adverse events (AEs) were monitored and classified based on the Common Terminology Criteria for Adverse Events (CTCAE) Version 5.0. Safety data were collected through physical examinations, laboratory tests, and imaging studies. Treatment-emergent adverse events (TEAEs) were categorized by frequency and severity. The data were analyzed according to MedDRA System Organ Class (SOC) and Preferred Term (PT).

### Efficacy assessment

Efficacy was assessed in phase IIa only. Key measures included changes in ICQ, PUF, nighttime voiding frequency, and the number and size of Hunner lesions as observed via cystoscopy at 1, 3, and 6 months post-administration. The global response assessment (GRA) was used to evaluate overall treatment response based on a 7-point scale, with “moderately improved” or “markedly improved” considered a positive outcome.

### Statistical analysis

All analyses were performed using SAS (version 9.4 or higher). Efficacy was evaluated using the last observation carried forward (LOCF) method for participants who completed treatment. Statistical significance was assessed using 2-sided tests with a significance level of 5%. Descriptive statistics were used for continuous variables, while percentages were used for categorical data. Where necessary, 95% confidence intervals were also calculated.

## Results

### Study participants

**Figure 2.**
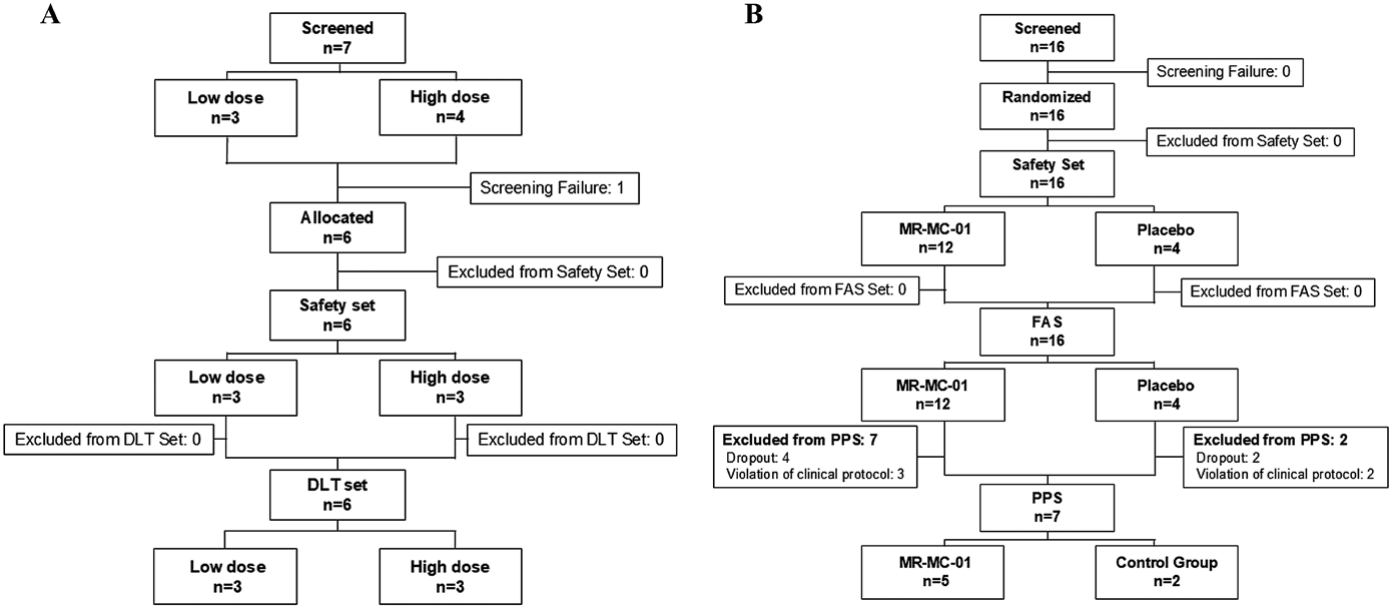
Disposition of subjects in phase 1 and phase Ⅱa studies. (A) Phase I clinical trial: open label. **(B)** Phase IIa clinical trial: double-blind, randomized, parallel. DLT: dose-limiting toxicity, any adverse event classified as Grade 3 or higher that limits the dose of a treatment. FAS: full analysis set, A group of participants that includes all individuals who were randomized and received treatment, regardless of adherence to the protocol. PPS: Per-Protocol Set, A group of participants who completed the study according to the protocol, excluding those who deviated from the study procedures.

A total of 22 participants were enrolled, with 6 in phase I and 16 in phase IIa. Of the 16 phase IIa participants, 12 were assigned to the MR-MC-01 group and 4 to the placebo group. Six participants withdrew from the study (4 from the MR-MC-01 group and 2 from the placebo group), but none of the withdrawals were due to adverse effects (Figure 2). Baseline characteristics were similar between the groups (Table 1).

**Table 1.** Demographic and clinical characteristics of the participants at baseline ^a^.

	Phase Ⅰ		Phase Ⅱa ^b^	
Characteristic	Low-dose (*n* = 3)	High-dose (*n* = 3)	MR-MC-01 (*n* = 12)	Placebo (*n* = 4)
Age (years)	69.7 ± 4.2	68.7 ± 6.5	64.4 ± 6.5	60.8 ± 5.0
Gender				
Male	0 (0)	1 (33.3)	3 (25)	2 (50)
Female	3 (100)	2 (66.7)	9 (75)	2 (50)
BMI (kg/m²) ^c^	24.2 ± 1.4	23.6 ± 2.5	22.5 ± 3.1	23.0 ± 3.2
Symptom duration (years)	5.3 ± 1.5	6.3 ± 2.9	6.5 ± 4.5	8.8 ± 1.7
10-point VAS ^d^	NA ^b^	NA ^b^	6.2 ± 1.3	4.3 ± 0.5
ICQ total score ^e^	NA ^b^	NA ^b^	29.8 ± 4.8	25.5 ± 6.4
PUF total score ^f^	NA ^b^	NA ^b^	22.6 ± 4.0	20.5 ± 5.7

^a^Data are reported as *n* (%) or mean ± SD.

^b^The evaluation of its effectiveness was conducted only in phase IIa.

^c^Body mass index, is calculated by dividing a person’s weight in kilograms by their height in meters squared.

^d^The 10-point Visual Analog Scale, is a subjective measure used to assess pain intensity. It consists of a line where one end represents “no pain” (0) and the other end represents “worst imaginable pain.”^10^.

^e^The interstitial cystitis questionnaire, is a tool used to assess the symptoms and the impact of interstitial cystitis on patients’ lives.

^f^Pain and urgency frequency, is a tool used to assess the symptoms and severity of interstitial cystitis in patients.

### Safety evaluations

In phase I, all 6 participants experienced adverse events, but none were related to the drug, and no dose-limiting toxicities (DLTs) were observed. The most common adverse events were mild and included bladder pain and procedural discomfort, all of which resolved without intervention.

In phase IIa, 5 treatment-emergent adverse events (TEAEs) occurred in the MR-MC-01 group, and 4 in the placebo group. No serious drug-related adverse events were observed. One serious adverse event, a case of transitional cell carcinoma in the placebo group, was treated successfully with transurethral resection and was unrelated to the investigational product. Importantly, no acute adverse events were reported following MR-MC-01 administration (Table 2, Table 3, Table 4).

**Table 2.** Summary of overall adverse event incidence.

	Phase Ⅰ		Phase Ⅱa	
	Low-dose (*n* = 3)	High-dose (*n* = 3)	MR-MC-01 (*n* = 12)	Placebo (*n* = 4)
TEAE				
N (%)	3 (100)	3 (100)	4 (33.3)	3 (75)
Event	5	3	5	4
95% CI ^a^	29.24, 100.00	29.24, 100.00	9.92, 65.11	19.41, 99.37
ADR				
*N* (%)	0 (0)	0 (0)	0 (0)	0 (0)
Event	–	–	–	–
95% CI ^a^	0.00, 70.76	0.00, 70.76	0.00, 26.46	0.00, 60.24
SAE				
*N* (%)	0 (0)	0 (0)	0 (0)	1 (25)
Event	–	–	–	1
95% CI ^a^	0.00, 70.76	0.00, 70.76	0.00, 26.46	0.63. 80.59
Acute AE				
*N* (%)	1 (33.3)	0 (0)	0 (0)	0 (0)
Event	1	–	–	–
95% CI ^a^	0.84, 90.57	0.00, 70.76	0.00, 26.46	0.00, 60.24

^a^Clopper-Pearson method.

**Table 3.** Summary of adverse events by SOC and PT for phase I ^a^

	Phase Ⅰ	
System organ class ^b^ Preferred term	Low-dose (*n* = 3)	High-dose (*n* = 3)
Injury, poisoning, and procedural complications	1 (33.33)	1 (33.33)
Post procedural hemorrhage	1 (33.33)	0 (0.00)
Procedural pain	0 (0.00)	1 (33.33)
Renal and urinary disorders	2 (66.67)	0 (0.00)
Bladder pain	2 (66.67)	0 (0.00)
Gastrointestinal disorders	1 (33.33)	0 (0.00)
Rectal spasm	1 (33.33)	0 (0.00)
Infections and infestations	0 (0.00)	1 (33.33)
Cystitis	0 (0.00)	1 (33.33)
Reproductive system and breast disorders	1 (33.33)	0 (0.00)
Vulval edema	1 (33.33)	0 (0.00)
Skin and subcutaneous tissue disorders	0 (0.00)	1 (33.33)
Vitiligo	0 (0.00)	1 (33.33)

^a^Data are reported as *n* (%).

^b^MedDRA(v26.0).

**Table 4.** Summary of adverse events by SOC and PT for phase II Ta ^a^

	Phase Ⅱa	
System organ class ^b^ Preferred Term	MR-MC-01 (*n* = 12)	Placebo (*n* = 4)
Renal and urinary disorders	1 (8.33)	2 (50.00)
Cystitis ulcerative	0 (0.00)	2 (50.00)
Hematuria	1 (8.33)	0 (0.00)
Infections and infestations	1 (8.33)	1 (25.00)
Genital herpes	0 (0.00)	1 (25.00)
Urinary tract infection	1 (8.33)	0 (0.00)
Injury, poisoning and procedural complications	2 (16.67)	0 (0.00)
Post procedural haemorrhage	1 (8.33)	0 (0.00)
Procedural pain	1 (8.33)	0 (0.00)
Neoplasms benign, malignant and unspecified (incl cysts and polyps)	0 (0.00)	1 (25.00)
Transitional cell carcinoma	0 (0.00)	1 (25.00)
Skin and subcutaneous tissue disorders	1 (8.33)	0 (0.00)
Rash	1 (8.33)	0 (0.00)

^a^Data are reported as *n* (%).

^b^MedDRA(v26.0).

### Clinical outcome evaluations

#### ICQ and PUF scores

**Figure 3.**
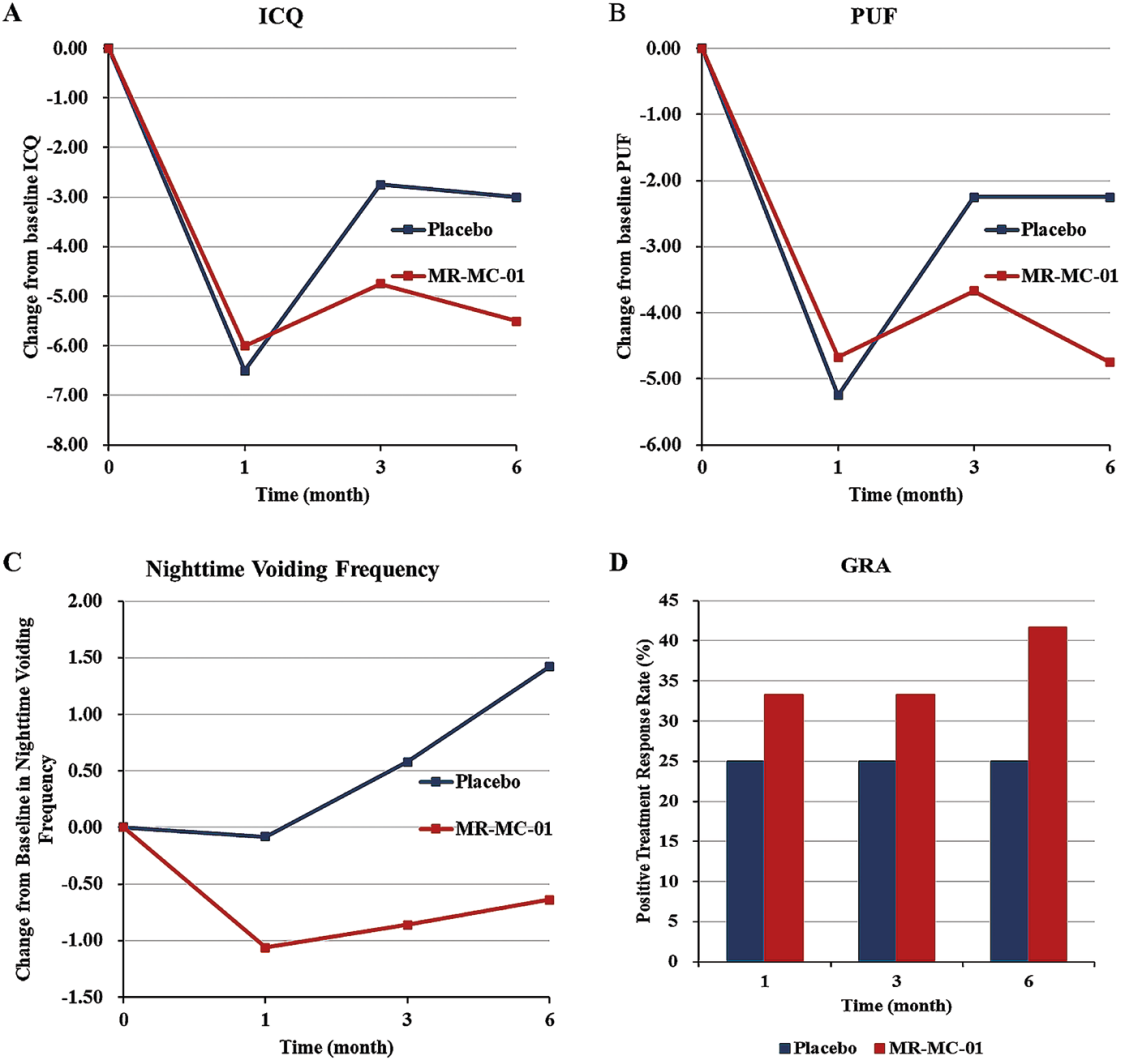
Change from baseline in clinical outcome measures comparing MR-MC-01 with placebo. (A) Total score of the interstitial cystitis questionnaire (ICQ), (B) Total score of the pain and urgency frequency (PUF), (C) average nighttime voiding frequency over three days, (D) global response assessment (GRA).

At 6 months, the MR-MC-01 group demonstrated significant improvements in ICQ and PUF scores compared to the placebo group. The MR-MC-01 group showed a mean reduction of -5.50 ± 6.93 points in ICQ, compared to −3.00 ± 7.35 in the placebo group. For PUF scores, the MR-MC-01 group saw a reduction of −4.75 ± 5.53 points, while the placebo group showed a reduction of only −2.25 ± 4.43 points (Table 5, Supplementary Tables 2, 3; Figure 3A, B). These improvements indicate that MR-MC-01 provided a more pronounced relief of IC symptoms compared to placebo.

**Table 5.** Clinical outcome measures improvement from baseline in MR-MC-01 group compared with the placebo group ^a^.

	Phase Ⅱa			
	MR-MC-01 (*n* = 12)	Placebo (*n* = 4)	Difference	*P value* ^b^
ICQ total score				
1 month	−6.00 ± 5.39	−6.50 ± 10.47	−0.50	0.93
3 months	−4.75 ± 6.18	−2.75 ± 6.70	2.00	0.60
6 months	−5.50 ± 6.93	−3.00 ± 7.35	2.50	0.56
PUF total score				
1 month	−4.67 ± 4.60	−5.25 ± 5.19	−0.58	0.84
3 months	−3.67 ± 4.87	−2.25 ± 4.43	1.42	0.30
6 months	−4.75 ± 5.53	−2.25 ± 4.43	2.50	0.18
Nighttime voiding frequency (AVE, for 3 days)				
1 month	−1.06 ± 1.01	−0.08 ± 1.20	0.98	0.16
3 months	−0.86 ± 1.11	0.58 ± 0.79	1.44	*
6 months	−0.64 ± 1.20	1.42 ± 1.10	2.06	**
GRA ^c^				
1 month	33.33	25	−–	–
3 months	33.33	25	–	–
6 months	41.67	25	–	–

^a^Data are reported mean ± SD.

^b^ *t*-Test between 2 groups (* *P* < .05, ** *P* < .01).

^c^Global response assessment (GRA) is a tool used to evaluate the overall improvement in a patient’s condition following treatment. Patients rated as “moderately improved” or “markedly improved” are considered to have experienced a positive treatment effect, and the percentage of participants showing positive treatment effects is reported.

#### Cystoscopy results

**Figure 4.**
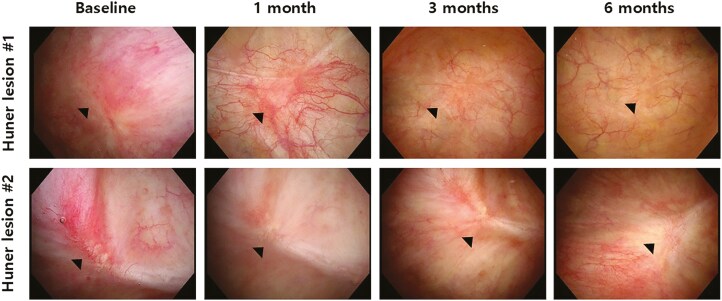
Sequential cystoscopy images of Hunner lesions in a patient receiving high‐dose MR-MC-01 (5 × 10^7^ cells). Images were taken at baseline, 1 month, 3 months, and 6 months. Black arrows indicate the lesion boundaries and tissue changes, demonstrating a progressive reduction and eventual disappearance of the lesions.

Eight out of 12 patients in the MR-MC-01 group exhibited a reduction or complete resolution of Hunner lesions at 6 months, compared to no significant changes in the placebo group. This suggests that MR-MC-01 may facilitate the healing or remission of bladder lesions in IC patients, offering a potential therapeutic advantage over standard care (Table 6, Figure 4).

**Table 6.** Changes in Hunner lesions over time in the MR-MC-01 treatment group (phase Ⅱa).

No.	After MR-MC-01 treatment		
	1 month	3 Months	6 Months
1	No change	Reduction in size	No change
2	Reduction in size	No change	No change
3	No change	Increase in size	No change
4	Lesion resolved	Lesion resolved	Lesion resolved
5	Lesion resolved	ND	No change
6	No change	ND	ND
7	No change	ND	ND
8	No change	No change	No change
9	Reduction in size	No change	ND
10	Reduction in size	Lesion resolved	No change
11	Lesion resolved	Lesion resolved	Lesion formation
12	No change	No change	Lesion resolved

#### Nighttime voiding frequency

A significant decrease in nighttime voiding frequency was observed in the MR-MC-01 group at both 3 and 6 months. At 6 months, the MR-MC-01 group showed a mean change of −0.64 ± 1.20 voids compared to an increase of +1.42 ± 1.10 voids in the placebo group. This reduction in nocturia reflects a meaningful improvement in bladder function, contributing to enhanced quality of life for patients (Table 5, Figure 3C).

#### GRA

The GRA further supported the efficacy of MR-MC-01. At 6 months, 41.67% of patients in the MR-MC-01 group reported moderate to marked improvement, compared to only 25% in the placebo group. This improvement suggests that MR-MC-01 may offer substantial benefits in symptom relief for patients with IC, particularly those unresponsive to conventional therapies (Table 5, Figure 3D).

## Discussion

This study aimed to evaluate the safety and efficacy of MR-MC-01, a cell therapy derived from human embryonic stem cells, in patients with IC who had not responded to conventional treatments. The results indicated that MR-MC-01 is both safe and effective, showing significant improvements in symptoms and quality of life for these patients.

Phase I of the trial revealed no dose-limiting toxicities or serious adverse events. In Phase IIa, the MR-MC-01 group showed notable reductions in the ICQ and PUF scores, consistent with initial clinical findings.^19^ The efficacy outcomes demonstrated a noteworthy reduction in the ICQ total score and PUF score among participants in the MR-MC-01 group. Furthermore, improvements were observed in the GRA, further validating the treatment’s positive impact. Particularly, a significant portion of the MR-MC-01 group experienced either a reduction or complete resolution of Hunner lesions after 6 months of treatment. This was accompanied by improvements in nighttime voiding frequency, an important factor for assessing patient comfort and overall bladder function. These results align with earlier trials using MR-MC-01.^19^

Cystoscopy results showed that 8 out of 12 patients in the MR-MC-01 group had reductions or complete resolution of Hunner lesions. This suggests that MR-MC-01 not only provides symptomatic relief but also promotes structural recovery of the bladder, which may decrease urinary frequency and urgency. The resolution of Hunner lesions implies a return to a healthier bladder state, reducing inflammation and restoring function. While the presence of Hunner lesions has been debated as a direct marker of IC severity, the results here suggest their resolution correlates with symptomatic improvement.^23-26^

Furthermore, the MR-MC-01 group demonstrated consistent improvements in ICQ and PUF scores at 3 and 6 months compared to the placebo group, providing strong evidence of MR-MC-01’s efficacy in treating IC. These outcomes underscore the therapy’s role not only in symptom management but also in potentially regenerating bladder tissue. This aligns with preclinical data, which suggest that mesenchymal stem cells (MSCs) may contribute to bladder structure restoration by differentiating into endothelial and epithelial cells. The WNT and IGF signaling pathways, known to promote tissue regeneration, may also play a role.^22^

One of the most significant clinical findings was the improvement in nighttime voiding frequency, which substantially improved patients’ quality of life. At 3 and 6 months, the MR-MC-01 group showed meaningful reductions in nighttime voids, while the placebo group exhibited an increase. This highlights the therapeutic benefits of MR-MC-01 in alleviating a key symptom of IC.

The GRA supported these results, with 41.67% of the MR-MC-01 group reporting meaningful improvement at 6 months, compared to 25% in the placebo group. This suggests sustained benefits over time, consistent with preclinical studies showing the long-term efficacy of stem cell-based therapies.^21,22^

This study has several limitations. First, pain assessment is inherently subjective, and individual differences—such as sex, race/ethnicity, and age—can influence pain perception, tolerance, and responses to treatment. In this trial, no significant improvement in VAS scores was observed at any point, likely due to the subjective nature of pain reporting. The VAS, commonly used for pain assessment in interstitial cystitis, may not fully capture treatment effects, suggesting the need for more objective tools to evaluate pain in future studies.

Second, while the study demonstrated positive trends in efficacy, not all outcomes reached statistical significance. The relatively small sample size, typical for a Phase I/IIa trial, may have limited the study’s ability to detect meaningful differences between the treatment and placebo groups. Additionally, the lack of diversity in the study population could limit the generalizability of the findings. Future studies should include larger and more diverse populations to ensure the results are broadly applicable.

While our findings demonstrate promising safety and efficacy outcomes at 6 months, long‐term durability of MR-MC-01 requires further investigation. To address this, we have initiated a 12‐month follow‐up with current participants, focusing on sustained symptom relief, late‐onset adverse events, and any relapse of Hunner lesions. In addition, we are planning a long‐term observational registry to capture data beyond one year and evaluate whether any supplemental or repeat treatment might be beneficial. These extended studies will provide more comprehensive insights into MR-MC-01’s potential for durable clinical benefits.

In addition, our sample size was relatively small, limiting the power to detect certain outcomes. To confirm the generalizability and robustness of our results, we are designing a larger, multi‐center phase 2b trial that will include a more diverse patient population across multiple institutions. Pending positive results from this trial, a phase 3 study is also being planned to further validate efficacy, compile extensive safety data, and potentially support regulatory approval. These planned trials will help clarify the translational impact of MR-MC-01 and strengthen its therapeutic position in managing IC.

Finally, a detailed analysis of the underlying mechanisms will help clarify how MR-MC-01 interacts with the inflammatory pathways involved in IC. Future studies should include patients with varying disease severity and underlying causes to gain a comprehensive understanding of the treatment’s potential.

Despite these limitations, this study provides strong evidence that MR-MC-01 is a promising and safe treatment option for interstitial cystitis, particularly for patients unresponsive to existing therapies. By addressing both symptoms and underlying disease mechanisms, MR-MC-01 offers a valuable approach that could pave the way for more effective treatments for chronic conditions like IC.

## Conclusion

MR-MC-01 shows significant potential as a safe and effective treatment for patients with interstitial cystitis who have exhausted other options. The study highlights its benefits for symptom relief and quality of life improvement, supported by favorable safety and efficacy data. Future research should explore the underlying mechanisms and focus on larger, multicenter trials to confirm these findings and optimize treatment protocols.

## Supplementary Material

szaf018_suppl_Supplementary_Tables

## Data Availability

The data underlying this article cannot be shared publicly due to the privacy of individuals who participated in the clinical study. De-identified data may be available from the corresponding author upon reasonable request and with appropriate institutional approvals.

## References

[1] Keller JJ , Chen YK, Lin HC. Comorbidities of bladder pain syndrome/interstitial cystitis: a population-based study. BJU Int. 2012;110:E903-E909. https://doi.org/10.1111/j.1464-410X.2012.11539.x 23020942

[2] Birder L , Andersson KE. Animal Modelling of Interstitial Cystitis/Bladder Pain Syndrome. Int Neurourol J. 2018;22:S3-S9. https://doi.org/10.5213/inj.1835062.531 29385788 PMC5798638

[3] Giannantoni A , Bini V, Dmochowski R, et al Contemporary management of the painful bladder: a systematic review. Eur Urol. 2012;61:29-53. https://doi.org/10.1016/j.eururo.2011.07.069 21920661

[4] Andersson KE , Birder L. Current pharmacologic approaches in painful bladder research: an update. Int Neurourol J. 2017;21:235-242. https://doi.org/10.5213/inj.1735022.511 29298474 PMC5756823

[5] Bosch PCA. Randomized, double-blind, placebo-controlled trial of certolizumab pegol in women with refractory interstitial cystitis/bladder pain syndrome. Eur Urol. 2018;74:623-630. 30072210 10.1016/j.eururo.2018.07.026

[6] Colaco MA , Evans RJ. Current recommendations for bladder instillation therapy in the treatment of interstitial cystitis/bladder pain syndrome. Curr Urol Rep. 2013;14:442-447. https://doi.org/10.1007/s11934-013-0369-y 24101384

[7] Kabay S , Kabay SC, Sevim M. First-line treatment posterior tibial nerve stimulation in patients with interstitial cystitis/bladder pain syndrome. Cent European J Urol. 2021;74:208-214. https://doi.org/10.5173/ceju.2021.0372 PMC8318015 34336240

[8] Hillelsohn JH , Rais-Bahrami S, Friedlander JI, et al Fulguration for Hunner ulcers: long-term clinical outcomes. J Urol. 2012;188:2238-2241. https://doi.org/10.1016/j.juro.2012.08.013 23083651

[9] Ko KJ , Cho WJ, Lee YS, et al Comparison of the efficacy between transurethral coagulation and transurethral resection of hunner lesion in interstitial cystitis/bladder pain syndrome patients: a prospective randomized controlled trial. Eur Urol. 2020;77:644-651. https://doi.org/10.1016/j.eururo.2020.01.002 31959549

[10] Clemens JQ , Erickson DR, Varela NP, Lai HH. Diagnosis and Treatment of interstitial cystitis/bladder pain syndrome. J Urol. 2022;208:34-42. https://doi.org/10.1097/JU.0000000000002756 35536143

[11] Chen JL , Kuo HC. Clinical application of intravesical botulinum toxin type A for overactive bladder and interstitial cystitis. Investig Clin Urol. 2020;61:S33-S42. https://doi.org/10.4111/icu.2020.61.S1.S33 PMC7004832 32055752

[12] Pinto R , Lopes T, Frias B, et al Trigonal injection of botulinum toxin A in patients with refractory bladder pain syndrome/interstitial cystitis. Eur Urol. 2010;58:360-365. https://doi.org/10.1016/j.eururo.2010.02.031 20227820

[13] Chermansky CJ , Guirguis MO. Pharmacologic management of interstitial cystitis/bladder pain syndrome. Urol Clin North Am. 2022;49:273-282. https://doi.org/10.1016/j.ucl.2022.01.003 35428433

[14] Hickson LJ , Eirin A, Lerman LO. Challenges and opportunities for stem cell therapy in patients with chronic kidney disease. Kidney Int. 2016;89:767-778. https://doi.org/10.1016/j.kint.2015.11.023 26924058 PMC4801657

[15] Kim A , Hoe KO, Shin JH, Choo MS. Evaluation of the incidence and risk factors associated with persistent frequency in interstitial cystitis/bladder pain syndrome and the efficacy of antimuscarinic treatment. Investig Clin Urol. 2017;58:353-358. https://doi.org/10.4111/icu.2017.58.5.353 PMC5577332 28868507

[16] Cheng J , Zheng Z, Tang W, et al A new strategy for stem cells therapy for erectile dysfunction: adipose-derived stem cells transfect Neuregulin-1 gene through superparamagnetic iron oxide nanoparticles. Investig Clin Urol. 2022;63:359-367. https://doi.org/10.4111/icu.20220016 PMC9091825 35534221

[17] Kim MY , Jo MS, Choi SG, et al Repeated injections of mesenchymal stem cell-derived exosomes ameliorate erectile dysfunction in a cavernous nerve injury rat model. World J Mens Health. 2024;42:787-796. https://doi.org/10.5534/wjmh.230218 38311373 PMC11439812

[18] Williams JK , Mariya S, Suparto I, Lankford SS, Andersson KE. Cell versus chemokine therapy effects on cell mobilization to chronically dysfunctional urinary sphincters of nonhuman primates. Int Neurourol J. 2018;22:260-267. https://doi.org/10.5213/inj.1836126.063 30599497 PMC6312977

[19] Shin JH , Ryu CM, Yu HY, et al Safety of human embryonic stem cell-derived mesenchymal stem cells for treating interstitial cystitis: a phase i study. Stem Cells Transl Med. 2022;11:1010-1020. https://doi.org/10.1093/stcltm/szac065 36069837 PMC9585946

[20] Hong KS , Bae D, Choi Y, et al A porous membrane-mediated isolation of mesenchymal stem cells from human embryonic stem cells. Tissue Eng Part C Methods. 2015;21:322-329. https://doi.org/10.1089/ten.TEC.2014.0171 25190318 PMC4346605

[21] Kim A , Yu HY, Lim J, et al Improved efficacy and in vivo cellular properties of human embryonic stem cell derivative in a preclinical model of bladder pain syndrome. Sci Rep. 2017;7:8872. https://doi.org/10.1038/s41598-017-09330-x 28827631 PMC5567131

[22] Ryu C-M , Yu HY, Lee H-Y, et al Longitudinal intravital imaging of transplanted mesenchymal stem cells elucidates their functional integration and therapeutic potency in an animal model of interstitial cystitis/bladder pain syndrome. Theranostics. 2018;8:5610-5624. https://doi.org/10.7150/thno.27559 30555567 PMC6276303

[23] Akiyama Y , Homma Y, Maeda D. Pathology and terminology of interstitial cystitis/bladder pain syndrome: a review. Histol Histopathol. 2019;34:25-32. https://doi.org/10.14670/HH-18-028 30015351

[24] Maeda D , Akiyama Y, Morikawa T, et al Hunner-type (classic) interstitial cystitis: a distinct inflammatory disorder characterized by pancystitis, with frequent expansion of clonal b-cells and epithelial denudation. PLoS One. 2015;10:e0143316. https://doi.org/10.1371/journal.pone.0143316 26587589 PMC4654580

[25] Akiyama Y , Niimi A, Nomiya A, et al Extent of Hunner lesions: the relationships with symptom severity and clinical parameters in Hunner type interstitial cystitis patients. Neurourol Urodyn. 2018;37:1441-1447. https://doi.org/10.1002/nau.23467 29315774

[26] Tomaszewski JE , Landis JR, Russack V, et al; Interstitial Cystitis Database Study Group. Biopsy features are associated with primary symptoms in interstitial cystitis: results from the interstitial cystitis database study. Urology. 2001;57:67-81. https://doi.org/10.1016/s0090-4295(01)01166-9 11378053

