# Supplementary material for: A single-center, phase 1/2a trial of hESC-derived mesenchymal stem cells (MR-MC-01) for safety and efficacy in interstitial cystitis patients
Source: Stem Cells Transl Med. 2025 May 19;14(5):szaf018. doi: 10.1093/stcltm/szaf018 (PMC12087345; doi:10.1093/stcltm/szaf018)
Supplement: szaf018_suppl_Supplementary_Tables [file szaf018_suppl_supplementary_tables.pdf]

Supplementary Information

Title

A Single-Center, Phase 1/2a Trial of hESC-Derived Mesenchymal Stem Cells (MR-MC-01) for Safety and Efficacy in Interstitial Cystitis Patients

Running head: Stem Cell Trial for Interstitial Cystitis

Yoon Soo Kyung, MD, PhD[# 1, 2], Ki-Sung Hong, PhD[# 3], Hyung-Min Chung, PhD[# 3, 4], Jung Hyun Shin MD, PhD[5], Myung-Soo Choo MD, PhD[1,6], Eun-Young Kim, PhD[3], Jeong Min Shin, PhD[3], Ah Reum Kang, MS[3], Jin Won Seo, MS[3], Juhyun Park MD, PhD[1 *], Se-Pill Park, PhD[3,7 *]

[1]Department of Urology, Asan Medical Center, University of Ulsan College of Medicine, Seoul 05505, Korea

[2]Health Screening and Promotion Center, Asan Medical Center, University of Ulsan College of Medicine, Seoul 05505, Korea

[3]Mirae Cell Bio Co., Ltd., Seoul 04795, Korea.

[4]Department of Stem Cell Biology, School of Medicine, Konkuk University, Seoul 05029, Korea.

[5]Department of Urology, Ewha Womans University, Mokdong Hospital, Seoul 07985, Korea

[6]Doctor Joo Urology Clinic, Seoul 06060, Korea

[7]Department of Bio Medical Informatics, College of Applied Life Sciences, Jeju National University, Jeju 63243, Korea.

#Yoon Soo Kyung, Ki-Sung Hong and Hyung-Min Chung contributed equally to this work.

*Corresponding authors: Juhyun Park, MD, PhD, Department of Urology, Asan Medical Center, University of Ulsan College of Medicine, 88, Olympic-ro 43-gil, Songpa-gu, Seoul 05505, Korea. Email: urojpark@amc.seoul.kr; Se-Pill Park, PhD, Department of Bio Medical Informatics, College of Applied Life Sciences, Jeju National University, 102 Jejudaehak-ro, Jeju, Jeju Special Self-Governing Province 63243, Korea, Email: sppark@jejunu.ac.kr

Supplementary Information

Supplementary Figure Legends

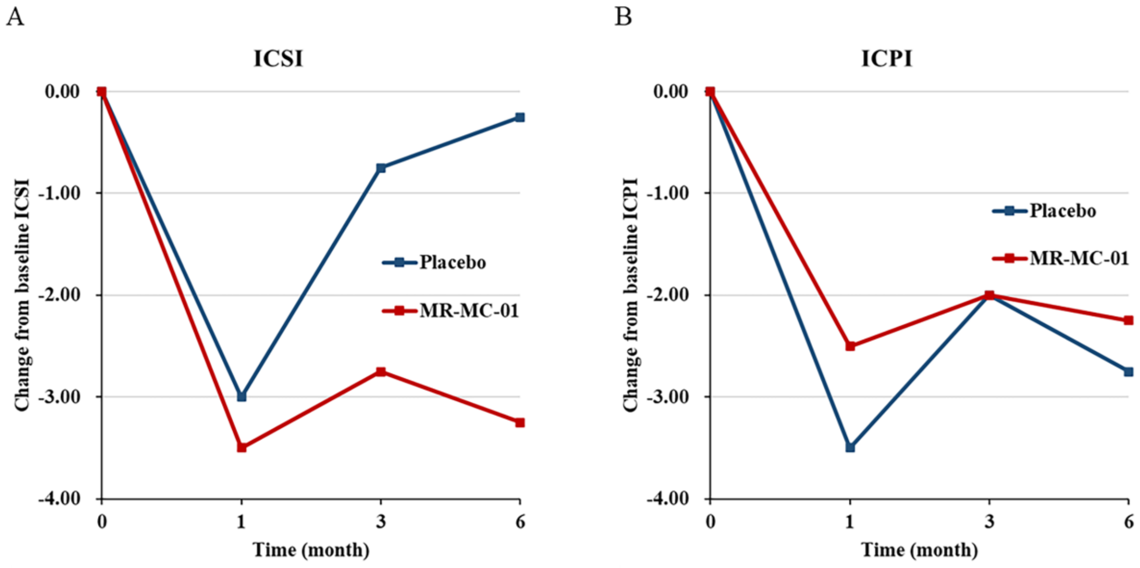

Supplementary Figure 1 The total score of the Interstitial Cystitis Questionnaire (ICQ) consists of ICSI and ICPI. (A) Interstitial Cystitis Symptom Index (ICSI) Score **(B)** Interstitial Cystitis Problem Index (ICPI) Score.

Supplementary Information

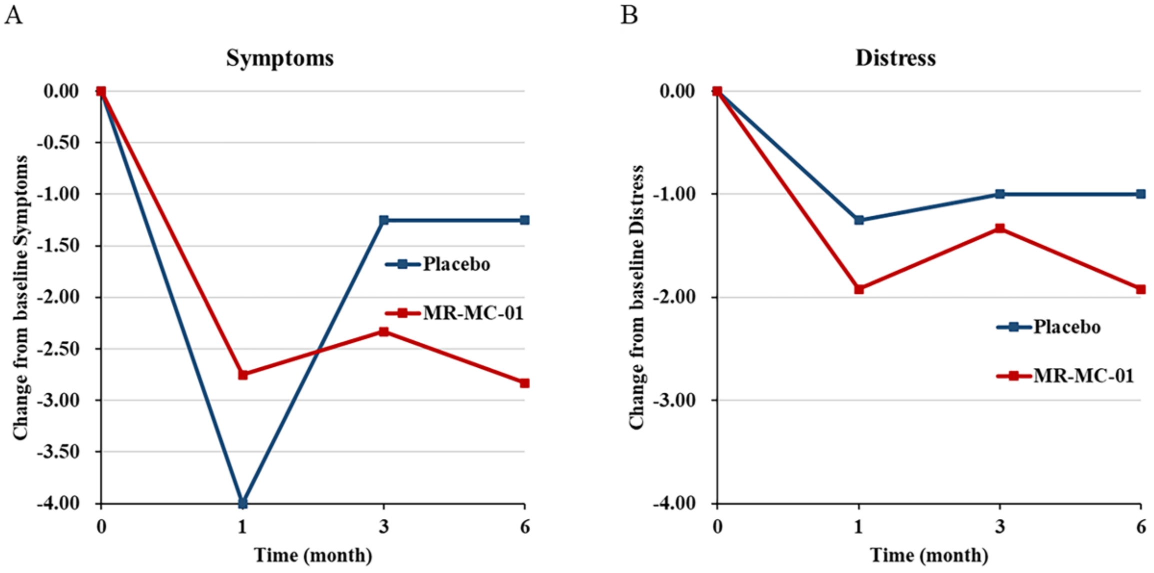

Supplementary Figure 2. The total score of Pain and Urgency Frequency (PUF) consists of symptom and distress components. (A) Symptom Score **(B)** Distress Score.

Supplementary Information

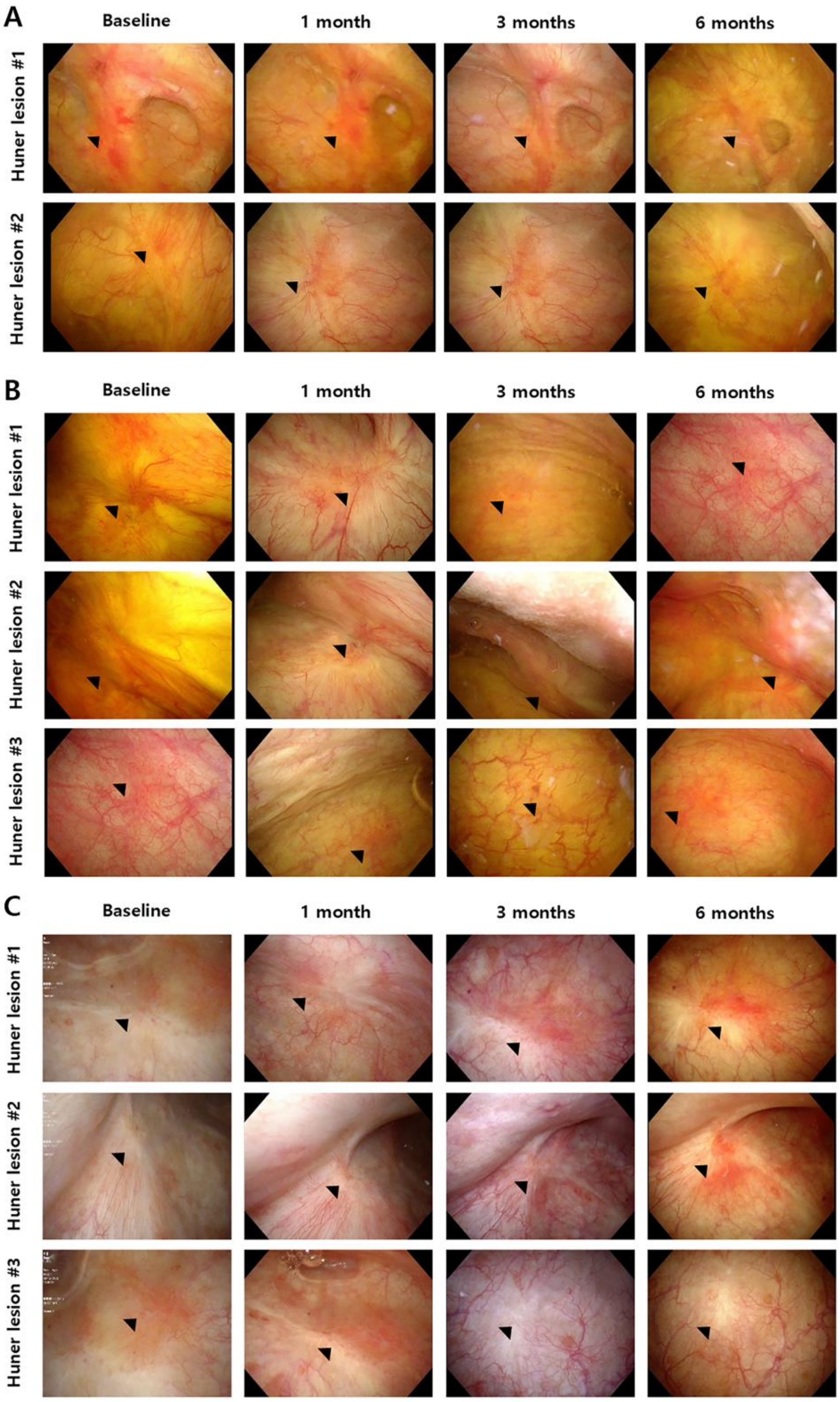

Supplementary Figure 3. Serial cystoscopic images of Hunner lesions in each patient. (A) Patient 1, (B) Patient 2, and (C) Patient 3. Hunner lesions in each patient at Baseline, 1 month, 3 months, and 6 months post-injection.

Supplementary Information

Black arrows indicate the lesion boundaries and tissue changes, demonstrating a progressive reduction and eventual disappearance of the lesions.

Supplementary Table 1. Inclusion and exclusion criteria.

Inclusion Criteria	Exclusion Criteria
1) Adult males and females aged 19 years or older<br>2) Those who have had the following symptoms and pain (not limited to) due to interstitial cystitis for more than 6 weeks<br>① Symptoms: Unpleasant sensation (pain, pressure, discomfort) felt in the bladder (pelvic area) related to the lower urinary tract without any infectious factor and other causes that cannot be identified, urinary urgency or frequent urination more than 10 times a day due to this unpleasant sensation<br>② Pain: Pain in the suprapubic area or the entire pelvis (urethra, vulva, vagina, rectum) or lower abdomen or lower back when urine is full, pain that occurs when the bladder is filled after eating certain foods, pain temporarily relieved during urination<br>3) Those who have Hunner lesion confirmed in cystoscopy performed within 1 month prior to screening<br>4) Those who did not respond to Pentosan polysulfate sodium treatment as a treatment for this indication according to the investigator's judgment or those who cannot be treated with Pentosan polysulfate sodium<br>5) Those who had pain evaluation due to interstitial cystitis at screening and baseline visits 10-point VAS (Visual analogue scale) 4 points or higher<br>6) At screening (in case of Phase 2a, up to baseline) O'Leary-Sant Interstitial Cystitis questionnaire (ICQ) total score at least 12 points or higher<br>7) At screening (in case of Phase 2a, up to baseline) PUF questionnaire total score at least 13 points or higher<br>8) Those who have heard the explanation of this clinical trial and voluntarily agreed in writing to participate	1) Those who have a history of stem cell treatment prior to screening<br>2) Those who have been diagnosed with recurrent urinary tract infection (recurrence more than twice in the 6 months prior to screening or more than three times in the previous year) or currently have a disease with symptoms similar to this indication, such as urinary tract infection, acute or chronic prostatitis, chronic pelvic pain syndrome, benign prostatic hyperplasia, vaginitis, urethritis, etc.<br>3) Those who the investigator determines that it will be difficult to participate in this clinical trial due to an anatomical abnormality of the lower urinary tract<br>4) Those who have been prescribed tricyclics for this indication within 2 weeks prior to screening If taking antidepressants (e.g., Pentosan polysulfate sodium, Amitriptyline HCl, etc.), Gabapentinoid drugs (e.g., Gabapentin, Pregabalin)<br>5) Those with the following past history in relation to this indication<br>① Received procedures or therapies within 6 months prior to screening that may affect the efficacy evaluation related to this indication<br>② Those with a history of urethral dilatation or urethrotomy within 3 months prior to screening<br>6) Those with a history of hysterectomy, urinary incontinence surgery, transvaginal surgery, pelvic organ prolapse surgery, vaginal delivery, or cesarean section within 6 months prior to screening<br>7) Those who were using an indwelling urinary catheter at the time of screening or those who repeatedly or habitually performed intermittent self-catheterization

Supplementary Information

Supplementary Table 2. ICQ total score (ICSI/ICPI): Comparison of MR-MC-01 Group and Placebo Group in Phase IIa [a]

	**Phase IIa**			
	**MR-MC-01 (n=12)**	**Placebo (n=4)**	**Difference**	***P value*** [b]
**ICSI (from ICQ)**				
**1 month**	-3.50 ± 2.97	-3.00 ± 5.83	0.50	0.87
**3 months**	-2.75 ± 2.93	-0.75 ± 4.03	2.00	0.19
**6 months**	-3.25 ± 4.07	-0.25 ± 4.11	3.00	*
**ICPI (from ICQ)**				
**1 month**	-2.50 ± 2.78	-3.50 ± 4.93	-1.00	0.70
**3 months**	-2.00 ± 3.44	-2.00 ± 3.56	0.00	1.00
**6 months**	-2.25 ± 3.52	-2.75 ± 3.30	-0.50	0.80

[a] Data are reported mean ± SD.

[b] t-test between two groups (* $p < 0.05$, ** $p < 0.01$).

Supplementary Information

Supplementary Table 3. PUF total score (Symptoms / Distress): Comparison of MR-MC-01 Group and Placebo Group in Phase IIa [a]

	Phase IIa			
	**MR-MC-01 (n=12)**	**Placebo (n=4)**	**Difference**	***P value*** [b]
**Symptoms (from PUF)**				
**1 month**	-2.75 ± 3.39	-4.00 ± 3.56	-1.25	0.54
**3 months**	-2.33 ± 3.28	-1.25 ± 2.63	1.08	0.25
**6 months**	-2.83 ± 3.97	-1.25 ± 2.63	1.58	0.2
**Distress (from PUF)**				
**1 month**	-1.92 ± 1.38	-1.25 ± 1.71	0.67	0.25
**3 months**	-1.33 ± 1.92	-1.00 ± 1.83	0.33	0.75
**6 months**	-1.93 ± 1.78	-1.00 ± 1.83	0.92	0.2

[a] Data are reported mean ± SD.

[b] t-test between two groups (* $p < 0.05$, ** $p < 0.01$).

Supplementary Information

Supplementary Table 4. Changes in Hunner Lesions Over Time in the MR-MC-01 Treatment Group (Phase 1)

No.	Dosage Administered	After MR-MC-01 Treatment (at 6 Months)
1	Low	No change
2	Low	No change
3	Low	No change
4	High	Reduction in size
5	High	No change
6	High	Lesion resolved
